# Behavioural and Cognitive Changes in Neurodegenerative Diseases and Brain Injury

**DOI:** 10.1155/2018/4935915

**Published:** 2018-07-25

**Authors:** Francesca Trojsi, Foteini Christidi, Raffaella Migliaccio, Hernando Santamaría-García, Gabriella Santangelo

**Affiliations:** ^1^Department of Medical, Surgical, Neurological, Metabolic and Aging Sciences, University of Campania “Luigi Vanvitelli”, Naples, Italy; ^2^First Department of Neurology, Aeginition Hospital, Medical School, National and Kapodistrian University of Athens, Athens, Greece; ^3^INSERM U 1127, CNRS UMR 7225, Sorbonne Universités, and Université Pierre et Marie Curie-Paris 6, UMRS 1127, Institut du Cerveau et de la Moelle Épinière (ICM), 75013 Paris, France; ^4^Centro de Memoria y Cognición Intellectus, Hospital Universitario San Ignacio, Pontificia Universidad Javeriana Bogotá, Bogotá, Colombia; ^5^Department of Psychology, University of Campania “Luigi Vanvitelli”, Caserta, Italy

Neurodegenerative diseases (NDs) are a wide range of neurological disorders affecting distinct subsets of neurons in specific anatomic systems, resulting in variable disease phenotypes [1]. Conventional clinicopathological approach allowed to identify several spectra of neurodegeneration, such as those associated to tauopathies, synucleinopathies, and Tar-DNA binding protein (TDP) 43-proteinopathies. However, it has been increasingly appreciated that many NDs overlap at multiple levels, including genetic, molecular, and neuroimaging levels [2–7], thereby overcoming classification approaches based only on clinicopathological patterns [1]. In particular, with regard to clinical phenotypes, behavioural and cognitive changes, proven to impact negatively patients' quality of life and prognosis [8, 9], have been associated to a large array of NDs, although showing peculiar profiles across the different syndromes. Alzheimer's disease (AD) is usually characterized by a progressive amnestic syndrome in elderly subjects, although younger patients may also present an atypical, focal, clinical syndrome in which a single cognitive domain, not related to memory, is predominantly affected, such as logopenic variant of primary progressive aphasia (lvPPA) and posterior cortical atrophy (PCA) [10]. Behavioural changes are the clinical core of the behavioural variant of frontotemporal dementia (bvFTD), ranging from apathy to disinhibition [11], but are also frequently reported in amyotrophic lateral sclerosis (ALS) [4] and parkinsonian plus syndromes (dementia with Lewy bodies, progressive supranuclear palsy, corticobasal syndrome, and Parkinson's disease—dementia) [12]. Specifically, impairments of primary motor and/or executive and language functions are more commonly reported in ALS and frontotemporal lobar degeneration (FTLD) [4, 11], while an increasing detection of both extrapyramidal symptoms and cognitive changes, including executive and language dysfunctions, limb apraxia, and visuoconstructive deficits, has been described within the parkinsonian plus syndromes [12]. Finally, ischemic/hemorrhagic stroke or traumatic brain injury (TBI) may be associated to behavioural and cognitive alterations similar to those described in NDs [8]. To note, molecular modifications, involving AD-related proteins (i.e., *β*-amyloid peptide, hyperphosphorylated tau protein, presenilins, apolipoproteins, and secretases), seem to play a pivotal role also on poststroke dementia and neuroinflammatory response to TBI [13].

On this background, this special issue addresses most recent insights into the field of neuropsychology of NDs, poststroke dementia, and TBI, from several points of view, ranging from the animal models of disease to potential and innovative therapeutic approaches on the neurodegenerative process.

With regard to animal models useful for investigating the role of physical exercise (PE) on counteracting stressors, L. Zhang et al. (“Effect of Voluntary Wheel Running on Striatal Dopamine Level and Neurocognitive Behaviors after Molar Loss in Rats”) addressed the effect of voluntary wheel running on striatal dopamine levels in Sprague-Dawley rats randomly divided into 4 groups (i.e., controls, molar loss group, 1-week PE before tooth loss group, and 4-week PE before tooth loss group). By comparing striatal levels of dopamine and performances at cognitive and behavioural tasks among the four groups, the two groups performing PE before tooth loss exhibited significantly increased striatal dopamine levels and improved neurobehavioural performances. These findings interestingly supported the positive effect of PE on cognition and emotion, emphasizing the yet recognized pivotal role of striatonigral pathway on behaviour and emotion regulation.

With regard to the clinical studies published in this special issue, the prospective investigation by F.G. van Rooij et al. (“Subjective Cognitive Impairment, Depressive Symptoms, and Fatigue after a TIA or Transient Neurological Attack: A Prospective Study”) explored the clinical course of 103 patients after transient ischemic attack (TIA) or nonfocal transient neurological attack (TNA), in terms of cognitive and behavioural changes, and investigated the longitudinal diffusion-weighted imaging (DWI) brain white matter alterations. Interestingly, subjective cognitive impairment and fatigue were more severe after six months only in DWI-positive patients, without showing differences between TIA and TNA, thereby confirming the concept that DWI hyperintensities are associated with permanent brain damage, cognitive decline, mood disorders, and fatigue in stroke patients.

With regard to cognitive assessment tools, C. Meyniel et al. (“COGEVIS: A New Scale to Evaluate Cognition in Patients with Visual Deficiency”) introduced a new evaluation tool, named COGEVIS (COGnitive Evaluation in VISual impairment), a set of neuropsychological tests conceived to assess the cognitive functions without the use of vision. COGEVIS was used to study the cognitive status of 38 visually impaired patients referred to the low vision rehabilitation (LVR) center of Sainte-Marie Hospital in Paris (France). COGEVIS was able to identify cognitive impairment with significant diagnostic value (area under the ROC curve of 0.84) and was also proposed to assess the cognitive evolution after LVR.

The Spanish/Colombian/Canadian group of R. Iodice et al. (“Sentence Context and Word-Picture Cued-Recall Paired-Associate Learning Procedure Boosts Recall in Normal and Mild Alzheimer's Disease Patients”) employed a word-picture paradigm to examine the effectiveness of combined pictorial illustrations and sentences to explore the word-recall performance in 18 healthy older adults (HOA) and 18 mild Alzheimer's disease (AD) patients. The authors revealed that, in both groups, pictures improved item recall compared to word condition such as sentences and that the HOA group performs better than mild AD group in all conditions. Moreover, word picture and sentence context were demonstrated to strengthen the encoding in the explicit memory task in both groups. These findings are consistent with the idea that older individuals, with and without dementia, are sensitive to the semantic constraints provided by a sentence context or picture. Moreover, they suggest future research lines on implementation of strategies to improve the memory of older people for verbalized instructions, in order to restore or potentiate sequential abilities in everyday life.

With regard to nonpharmacological interventions used for inducing neuroplasticity, P. Cruz Gonzalez et al. (“The Effects of Transcranial Direct Current Stimulation on the Cognitive Functions in Older Adults with Mild Cognitive Impairment: A Pilot Study”) aimed to investigate the effect of a combined approach, which associated transcranial direct current stimulation (tDCS) on the left dorsolateral prefrontal cortex and cognitive rehabilitation through cognitive stimulation (CS) programme, on cognitive performances of five patients with mild cognitive impairment (MCI). The authors revealed that tDCS together with CS improved performances in multiple cognitive domains showing better results than those achieved by using CS alone. These preliminary findings could give further clues to optimize therapeutic approaches by noninvasive brain stimulation.

With regard to advanced neuroimaging evidence, the study by P. Reyes et al. (“Functional Connectivity Changes in Behavioral, Semantic, and Nonfluent Variants of Frontotemporal Dementia”) investigated brain functional connectivity changes by resting state functional magnetic resonance imaging (RS-fMRI) in 76 patients exhibiting the three FTD variants (bvFTD, semantic, and nonfluent variants of primary progressive aphasia—svPPA and nfvPPA) in order to find specific connectivity alteration in each variant. The authors compared the whole brain functional connectivity and the topologic measures, such as global efficiency, degree, path length, and clustering, obtained in FTD patients with those measured in healthy controls and between the three variants. Both neuropsychological and neuroimaging results showed similarity among FTD variants. In particular, there were no differences between the PPA variants. Nevertheless, the nfvPPA variant showed loss of global efficiency compared to bvFTD, more severe than that exhibited by svPPA group compared with bvFTD. Conversely, the bvFTD group showed an extended bilateral disconnection between frontal and limbic hubs and the basal ganglia that seems to be associated with the disruption between affective and self-referential brain systems in bvFTD patients.

Finally, the review article, written by the guest editors team, on “Social Cognition Dysfunctions in Neurodegenerative Diseases: Neuroanatomical Correlates and Clinical Implications” widely addressed recent evidence regarding the impairment of brain networks related to emotion processing, theory of mind, and empathy. Moreover, the review article addressed the most updated evidence on clinical manifestations and assessment tools for evaluating social cognitive dysfunctions in most NDs, such as FTLD, amyotrophic lateral sclerosis (ALS), and Parkinson's and Alzheimer's diseases, prospecting potential benefits on patients' well-being, quality of life, and outcome derived from pharmacological and nonpharmacological therapeutic approaches to these deficits.

In conclusion, the substantial message of this special issue is that abnormal behaviour and cognitive changes, commonly observed in clinical practice, represent an important part of the core diagnostic criteria for many clinical disorders. Cognitive and behavioural alterations are a risk factor for morbidity and mortality through not adherence to treatments and social isolation [8, 9]. Pharmacological and also nonphamacological interventions, aimed to counteract the potential consequences related to cognitive and behavioural alterations, should be implemented in order to reduce the negative effects of such disabilities on patients' and their caregivers' well-being and quality of life.

## References

[1] Ahmed R. M., Devenney E. M., Irish M. (2016). Neuronal network disintegration: common pathways linking neurodegenerative diseases. *Journal of Neurology, Neurosurgery & Psychiatry*.

[2] Lomen-Hoerth C., Anderson T., Miller B. (2002). The overlap of amyotrophic lateral sclerosis and frontotemporal dementia. *Neurology*.

[3] Kobylecki C., Jones M., Thompson J. C. (2015). Cognitive-behavioural features of progressive supranuclear palsy syndrome overlap with frontotemporal dementia. *Journal of Neurology*.

[4] Strong M. J., Abrahams S., Goldstein L. H. (2017). Amyotrophic lateral sclerosis - frontotemporal spectrum disorder (ALS-FTSD): revised diagnostic criteria. *Amyotrophic lateral sclerosis and frontotemporal degeneration*.

[5] Heyburn L., Moussa C. E. (2017). TDP-43 in the spectrum of MND-FTLD pathologies. *Molecular and Cellular Neurosciences*.

[6] Giannini L. A. A., Irwin D. J., McMillan C. T. (2017). Clinical marker for Alzheimer disease pathology in logopenic primary progressive aphasia. *Neurology*.

[7] Peng C., Gathagan R. J., Lee V. M. (2018). Distinct *α*-synuclein strains and implications for heterogeneity among *α*-synucleinopathies. *Neurobiology of disease*.

[8] Douven E., Aalten P., Staals J. (2018). Co-occurrence of depressive symptoms and executive dysfunction after stroke: associations with brain pathology and prognosis. *Journal of Neurology, Neurosurgery & Psychiatry*.

[9] Hallikainen I., Hongisto K., Välimäki T., Hänninen T., Martikainen J., Koivisto A. M. (2018). The progression of neuropsychiatric symptoms in Alzheimer's disease during a five-year follow-up: Kuopio ALSOVA study. *Journal of Alzheimer's Disease*.

[10] Warren J. D., Fletcher P. D., Golden H. L. (2012). The paradox of syndromic diversity in Alzheimer disease. *Nature Reviews Neurology*.

[11] Hodges J. R., Piguet O. (2018). Progress and challenges in frontotemporal dementia research: a 20-year review. *Journal of Alzheimer's Disease*.

[12] Deutschländer A. B., Ross O. A., Dickson D. W., Wszolek Z. K. (2018). Atypical parkinsonian syndromes: a general neurologist’s perspective. *European Journal of Neurology*.

[13] Kokiko-Cochran O. N., Godbout J. P. (2018). The inflammatory continuum of traumatic brain injury and Alzheimer’s disease. *Frontiers in Immunology*.

